# Identification and genetic characterization of a distinct genotype of Puumala orthohantavirus in Hebei Province, China

**DOI:** 10.1371/journal.pntd.0014250

**Published:** 2026-05-11

**Authors:** Yanan Cai, Yamei Wei, Guoyi Du, Xinyang Zhang, Zhenkun Wang, Zhengguang Wang, Zhanying Han, Yanbo Zhang, Yonggang Xu, Xu Han, Jiandong Li, Qi Li

**Affiliations:** 1 School of Public Health, Hebei Medical University, Shijiazhuang, China; 2 Institute for Viral Disease Control and Prevention, Hebei Provincial Center for Disease Control and Prevention, Shijiazhuang, China; 3 Hebei Key Laboratory of Pathogens and Epidemiology of Infectious Diseases, Hebei Provincial Center for Disease Control and Prevention, Shijiazhuang, China; 4 Hebei Provincial Institute for Plague Control, Zhangjiakou, China; 5 School of Public Health, Hebei University, Baoding, China; 6 National Institute for Viral Disease Control and Prevention, Chinese Center for Disease Control and Prevention, Beijing, China; Faculty of Science, Ain Shams University (ASU), EGYPT

## Abstract

Orthohantavirus infections pose a significant threat to human health, while numerous orthohantaviruses have been identified, suspected viral infections remain undiagnosed in the world, which highlights the need for further identification and characterization of viruses circulating in humans and host animals. In this study, viral metagenomics was utilized to investigate orthohantaviruses present in tissue samples collected from rodents trapped at the Bashang Grassland of Hebei Province, China. A total of 145 wild rodents belonging to six species were captured in the study area, and 725 tissue samples (lung, liver, kidney, spleen, gut) were collected in 2024. A Puumala orthohantavirus (PUUV), named Guyuan strain, was identified in *Myodes rufocanus*, with a positive rate of 0.69%. The complete genomic sequences of the L, M, and S segments were obtained and confirmed by Sanger sequencing. Phylogenetic analysis of these genomic sequences with those of other orthohantavirus species showed that the L, M, and S segments clustered with PUUV genomic sequences, while sharing a nucleotide sequence similarity of 81.2%, 80.2%, and 84.3% with previously characterized reference viral strains Kitahiyama128L, Tobetsu_04, and Baltic/205 Cg, respectively. Amino acid homology analysis demonstrated that the sequences exhibited the highest identity to PUUV Hokkaido strain at a level of 95.4%, 94.6%, and 97.0% respectively. Viral particles were observed in lung and kidney tissues using transmission electron microscopy, and viral protein antigen was detected in viral RNA-positive lung, liver, and kidney tissues through immunofluorescence assay with antibodies against the PUUV nucleocapsid protein, thereby confirming the virus’s multiorgan tropism. The results demonstrated that a distinct genotype of PUUV was circulating in rodents in the study areas, which may have implications for zoonotic transmission surveillance and public health management in Hebei Province.

## Author summary

Rodents serve as the predominant natural reservoirs of orthohantaviruses and the source of human infection, and numerous rodent-borne orthohantaviruses can cause hemorrhagic fever with renal syndrome (HFRS). HFRS caused by Hantaan orthohantavirus (HTNV) and Seoul orthohantavirus (SEOV) represents a significant public health problem in Hebei Province, China. However, it remains unclear whether other orthohantaviruses are present in Hebei Province. In 2024, a total of 145 wild rodents were captured in the study area, from which 725 tissue samples (lung, liver, kidney, spleen, gut) were collected. We first identified a distinct genotype of PUUV in Guyuan County, located in the Bashang Grassland area of Hebei Province, China. Amino acid homology analysis revealed that the sequences of this PUUV genotype shared the highest identity with the PUUV Hokkaido strain. Special attention should be paid to the distinct genotype of PUUV circulating in *Myodes rufocanus* within the study area. Further studies are necessary to establish longitudinal surveillance of rodent populations to clarify the transmission dynamics and assess the pathogenicity of the newly identified virus.

## Introduction

The rapid expansion in the diversity of *Bunyavirales*, their heightened significance in human disease causation, and the continuous evolution of higher-order taxonomic principles have collectively driven the ongoing refinement and revision of their taxonomic system [1]. In 2024, the International Committee on the Taxonomy of Viruses (ICTV, https://talk.ictvonline.org) officially reclassified *Bunyavirales* to the class *Bunyaviricetes*. *Hantaviridae* is an important branch taxonomic lineage within the order *Elliovirales*, class *Bunyaviricetes*. A substantial number of orthohantavirus species are categorized in *Hantaviridae* and demonstrate a wide host range spanning vertebrates, invertebrates, and plants, with certain species exhibiting the potential for cross-species transmission [2].

As of November 2023, ICTV officially recognized the family *Hantaviridae* as comprising eight genera encompassing 54 identified orthohantaviruses, with orthohantavirus being the most speciose genus containing 35 of these species. Among that, there are at least 28 known orthohantaviruses to cause hemorrhagic fever with renal syndrome (HFRS) or hantavirus pulmonary syndrome (HPS) [3–6]. Key pathogenic orthohantaviruses implicated in human disease include HTNV, SEOV, PUUV, Dobrava-Belgrade orthohantavirus (DOBV), and Tula orthohantavirus (TULV), which are predominantly associated with HFRS. However, Andes orthohantavirus (ANDV), Sin Nombre orthohantavirus (SNV), and Bayou orthohantavirus (BAYV) are associated with HPS. Globally, the estimated annual cases of HFRS or HPS ranges from 150,000–200,000, with case fatality rates varying depending on the viral type. In Asia, Amur virus-associated HFRS demonstrates high clinical severity, with mortality rates as high as 15%, compared to approximately 1% for HTNV and SEOV infections. In the Americas, NDV and SNV have fatality rates of 30–50%, while PUUV in Europe demonstrate significantly lower mortality with 0.1-0.4% [7].

PUUV was first identified in bank voles (*Myodes glareolus*) from Puumala region of Finland in the 1980s [8], and has emerged as the primary etiological agent of orthohantavirus infections across Europe over the past few decades, accounting for approximately 3,100 cases annually from 2010 to 2020. Within PUUV variant, eight PUUV lineages have been detected widely across Europe and Russia, based on complete S segment sequences, each lineage comprising well-supported variant clusters with distinct geographic structuring [9]. Additionally, PUUV-like viruses, such as Hokkaido virus (HOKV) in Hokkaido, Japan [10], Muju virus (MUJV) in South Korea [11], and Fusong virus in Jilin Province, China [12], have been identified. PUUV does not impair the growth or reproduction of its host animals, but can cause nephropathia epidemica (NE) in humans, which represents a less severe and milder form of HFRS [13].

Recent studies have revealed that orthohantaviruses have broadened their host range beyond rodents, with the viruses identified in various non-rodent mammals, including shrews, moles, and bats [14–16]. Most orthohantaviruses are rodent-borne pathogens displaying a marked host specificity, typically associated with one or a restricted set of closely related reservoir species and conforming to the distribution of their respective hosts [13,17]. PUUV is primarily carried by bank vole, which belongs to the subfamily *Arvicolinae* of the family *Muridae.* Notably, genetically closely related PUUV strains from Asia (including Japan and China) have been identified in *Myodes glareolus* and *Myodes rutilus*; however, no confirmed evidence of human pathogenicity has been reported to date [18,19].

Northern Hebei Province, characterized by its plateaus and mountainous regions, is recognized as a biodiversity hotspot rich in wildlife resources and also serves as a source of various zoonotic diseases. In this study, we carried out comprehensive investigations in the Bashang Grassland and the surrounding mountainous areas of the province and identified a distinct genotype of PUUV through metagenomic and Sanger sequencing analyses. Using electron microscopy, real-time fluorescent quantitative PCR, and indirect immunofluorescence assay (IFA), we characterized this virus and confirmed its tropism for multiple organs, which revealed the circulation of this distinct PUUV genotype in rodents from the study areas.

## Materials and methods

### Ethics statement

This research, including the specimen collection and processing, was reviewed and approved by the Ethics Committee of Hebei Provincial Center for Disease Control and Prevention (HeBIRBs2025012).

### Sample collection

In 2024, wild rodents were captured across Bashang Grassland region and mountainous area of northern in Hebei Province. Wild rodents were captured using cage traps, with a total of 200 collapsible traps deployed at each sampling site, spaced at intervals of 5–10 meters. All traps were baited with raw peanut kernels. Throughout the sampling period, the traps remained open continuously and were checked early each morning. Captured rodents were first subjected to on-site morphological identification to confirm species and then anesthetized with ether prior to being euthanized by cervical dislocation. Organ samples (lung, liver, kidney, spleen, and gut) were aseptically collected. Each specimen was recorded with details of collection date, sampling location, habitat, and biological traits (species, age, and sex), then transferred into labeled cryovials, snap-frozen in liquid nitrogen, and cryopreserved at -80°C prior to further processing.

### Metagenomic sequencing and sequence assembly

Pooled Lung samples from wild rodents were thoroughly homogenized, followed by total RNA extraction and quantification using a Qubit 4. Construction of RNA libraries was performed with RNA Transposase-based Library Preparation Kit (Jiangsu Bioperfectus Technologies Co., Ltd., Taizhou, China). Illumina NovaSeq6000 instrumentation was utilized for high-throughput sequencing in PE 150 mode. Sequencing data underwent quality control via fastp v0.20.0 [20] with adapters and low-quality sequences being filtered out. SortMeRNA v2.0 was used to estimate ribosome fragment elimination. Sequencing reads were aligned to the host reference genomes. MetaSPAdes v3.13.1 and MEGAHIT v1.2.9 were employed for de novo assembly of host-depleted sequencing data, yielding contigs as output. Assembled contigs underwent classification into recognized viral orders and families through BLASTN (2.14.0) alignment against the NCBI nt database, while annotation was conducted via the diamond BLASTx program based on the NCBI virus database.

### Nucleic acid extraction

Each organ tissue specimen (30–50 mg) was homogenized in 1 mL PBS containing grinding beads within a 1.5 mL microcentrifuge tube using a high-throughput tissue homogenizer. The homogenate, ground at low temperature to achieve complete homogenization, was centrifuged at 12,000 ×g for 10 minutes. The resulting supernatant was then subjected to total RNA extraction using nucleic acid extraction and purification kit (Jiangsu Bioperfectus Technologies Co., Ltd., Taizhou, China).

### Mitochondrial COI Gene-Based Identification of Rodent Species

Rodent species were identified using DNA barcoding, an approach commonly employed in molecular biology, by amplifying the mitochondrial COI gene [21]. The PCR reaction mixture consisted of Taq Green Master Mix, DNA and COI primer pairs, designated as COI-F (CCTACTCGGCCATTTTACCTATG) and COI-R (ACTTCTGGGTGTCCGAAGAATCA). PCR amplification was initiated with a 2-minute incubation at 94°C, followed by 35 cycles (each consisting of 30 s at 94°C, 30 s at 60°C, and 1 min at 72°C), and concluded with a final extension at 72°C for 5 min. The resultant products were subjected to Sanger sequencing (Shanghai, China).

### Real-time fluorescence quantitative PCR (RT-qPCR)

Total RNA was tested using the Puumala virus nucleic acid detection kit (Guangzhou Slin Medical Technology Co., Ltd., Guangzhou, China) following the manufacturer’s protocol. For each sample, 5 µL of RNA was combined with PUUV primers and probes in a one-step RT-qPCR master mix. The cycling conditions were as follows: 15 min of reverse transcription at 50°C, 5 min of initial denaturation at 95°C, followed by 40 cycles comprising 3s at 95°C and 45s at 55°C.

### Whole genome sequencing

Fifteen pairs of specific primers were designed based on metagenomic assembly results for PCR amplification of the full-length novel PUUV genome (primer information is provided in S1 Table). The positive RNA samples were then reverse transcribed using SuperScript IV First-Strand Synthesis System (Thermo Fisher Scientific, Waltham, MA, USA), in accordance with the manufacturer’s protocols. First-strand cDNA synthesis proceeded with cycling parameters of 25°C for 10 min, 50°C for 10 min, and 85°C for 5 min. For the secondary PCR amplification, the reaction mixture contained 3 µL of the first-round product, 12.5 µL GoTaq Colorless Master Mix, 2 µL of primers, and 7.5 µL nuclease-free water. The thermal cycling protocol was as follows: 95°C for 5 min, followed by 35 cycles of 94°C for 30 s, 55°C for 45 s, and 72°C for 1 min, with a final extension at 72°C for 10 min. The 5’ and 3’ terminal sequences of the PUUV cDNA were amplified and sequenced using the SMARTer RACE 5’/3’ Kit (Takara Bio Inc., Japan), as described previously [22]. The PCR products were identified by an automated capillary electrophoresis system (Bioptic Technology Co., Ltd., Jiangsu, China) and subsequently submitted to Sangon Biotech (Shanghai, China) for Sanger sequencing.

### Transmission electron microscopy and indirect immunofluorescence observation

Lung, liver, and kidney tissues from wild rodents with positive and negative results were sectioned, and viral particles were visualized using transmission electron microscope (Leica Microsystems, Wetzlar, Germany) (detailed protocols can be found in S1 File). Positive tissue sections were further examined by immunofluorescence assay (IFA). Briefly, organ tissue specimens were fixed and sectioned into 4-μm slices using a KD-III cryocut microtome (Jinhua Kedi Instrument Co., Ltd., China) at −20°C, followed by fixation in cold acetone. The primary antibody used was an anti-Hantavirus Puumala mouse monoclonal antibody (Progen Biotechnik GmbH, Heidelberg, Germany), with the secondary antibody being a FITC-conjugated goat anti-human Fab segment antibody (Abcam, Cambridge, UK). Fluorescent staining was conducted on the specimens, and scattered, granular fluorescence within tissue sections was considered indicative of positive staining.

### Viral isolation

Organs including lung, liver, spleen, and kidney obtained from positive rodent samples were homogenized. The resulting homogenates were resuspended in sterile PBS, centrifuged at 12,000 ×g to collect the supernatants, which were subsequently filtered through a 0.22 μm filter and preserved for further use. Vero and A549 cells were grown in DMEM (Dulbecco’s modified Eagle’s medium), while Vero E6 and BHK-21 cells were maintained in MEM (minimum essential medium). A 10% fetal bovine serum (FBS) supplement was added to both media. After being rinsed with PBS, the cells were trypsinized, centrifuged, and then mixed with the filtrate. This mixture was re-centrifuged at 1000 ×g for 5 minutes before being transferred to culture flasks. The cells were subjected to medium change every 7 days and blind-passaged for three generations, with each generation lasting 21 days. Concurrently, cell morphology was observed, and the supernatant was aspirated during each medium change and passage for viral nucleic acid detection.

### Phylogenetic analyses

Sequences of the novel PUUV variant were aligned with GenBank reference sequences, chosen based on genomic integrity (encompassing full-length L, M, and S segments). Reference sequences included eight PUUV lineages in Eurasia: Alp-Adrian (ALAD),Central European (CE), Danish (DAN), Finnish (FIN), LAT, North-Scandinavian (N-SCA), South-Scandinavian (S-SCA), and Russian (RUS), PUUV-like variants—HOKV (JAP), MUJV (KR), Fusong virus(CHN), and other 25 *Hantaviridae* members. Phylogenetic analysis was performed using MEGA software (Version 11) [23] and IQ-TREE Web (http://iqtree.cibiv.univie.ac.at/) under the maximum-likelihood (ML) framework, following the Bayesian information criterion (BIC). BIC values were computed via IQ-TREE with bootstrap replication set to 1000 iterations [24]. Phylogenetic trees were visualized and adjusted via the Chiplot Web platform (https://www.chiplot.online/) [25].

## Results

### Identification of PUUV

In 2024, 145 wild rodents were sampled from the Bashang Grassland and mountainous areas of northern Hebei Province, North China. Samples were collected from four counties under the administrative jurisdictions of Zhangjiakou and Chengde cities: Guyuan, Fengning, Weichang, and Kuancheng. The sampled species included *Citellus, Rattus, Meriones, Myodes, Apodemus,* and *Mus*, with *Citellus* being the dominant species, representing 39.31% (57/145) of the total collected samples (Table 1). Nucleic acid extracted from pooled lung samples was subjected to metagenomic sequencing using Illumina technology. Using BLASTn alignment, de novo-assembled contigs displayed multiple genomic regions with high similarity to the PUUV genome. RT-qPCR and Sanger sequencing were used to further confirm positive samples.

**Table 1:** Composition of rodent species and detection of the PUUV Guyuan strain in rodent samples from different sampling sites.

Sampling position	Longitude, Latitude	Landforms	Rodent species *(Latin name)*	Family	Genus	Total number of different rodents	Number of positive rodents	Positive rodent for different rodent species	Total number of rodents	Total number of positives
Guyuan county	115.8346, 41.6486	Grassland	*Spermophilus dauricus*	*Sciuridae*	*Citellus*	25	0	0%	50	1
			*Rattus norvegicus*	*Muridae*	*Rattus*	12	0	0%		
			*Meriones unguiculatus*	*Cricetidae*	*Meriones*	8	0	0%		
			*Myodes rufocanus*	*Cricetidae*	*Myodes*	3	1	33.33%		
			*Apodemus agrarius*	*Muridae*	*Apodemus*	2	0	0%		
Fengning county	116.0478, 41.6490	Grassland	*Spermophilus dauricus*	*Sciuridae*	*Citellus*	17	0	0%	25	0
			*Rattus norvegicus*	*Muridae*	*Rattus*	8	0	0%		
Weichang county	117.7639, 41.9236	Grassland	*Spermophilus dauricus*	*Sciuridae*	*Citellus*	15	0	0%	25	0
			*Rattus norvegicus*	*Muridae*	*Rattus*	10	0	0%		
Kuancheng county	118.3279, 40.7438	Mountainous area	*Rattus norvegicus*	*Muridae*	*Rattus*	24	0	0%	45	0
			*Mus musculus*	*Muridae*	*Mus*	21	0	0%		

**Fig 1:**
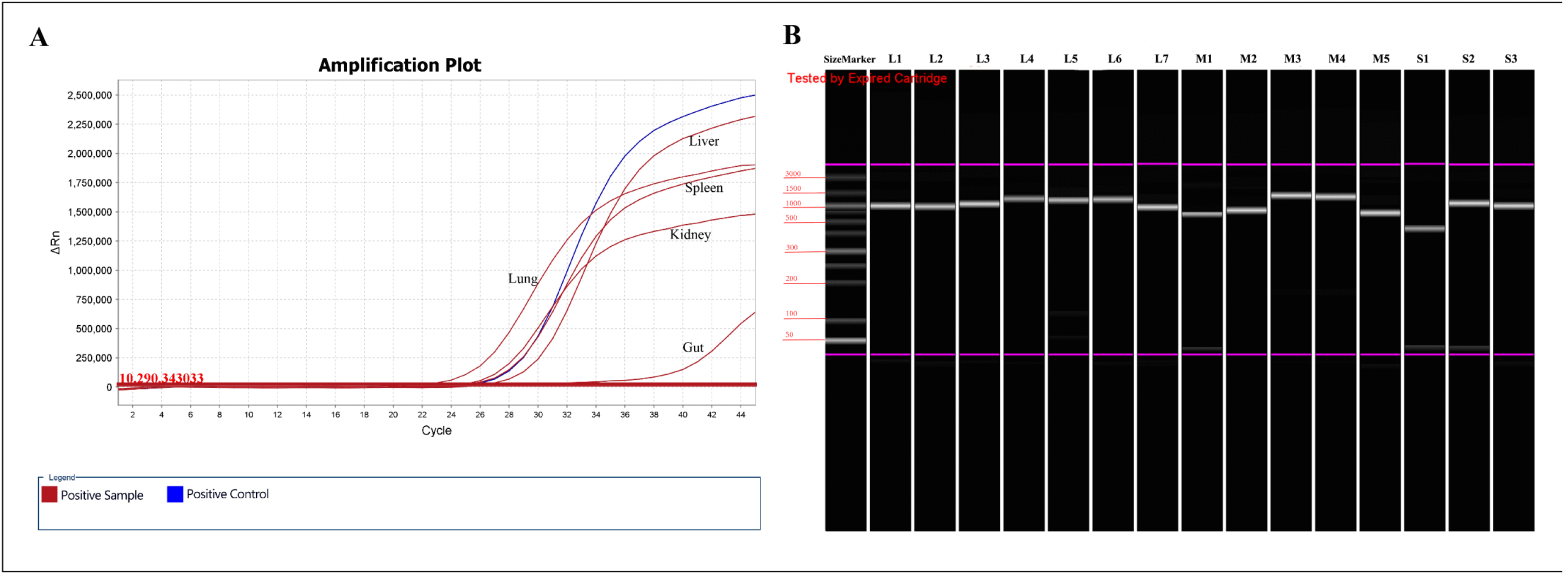
Virus detection results. **(A)** RT-qPCR detection for the PUUV Guyuan strain in different tissues. (Note: the red curve represents the positive sample; the blue curve represents the positive control.) **(B)** Capillary electrophoresis results of the full-genome amplification products of the PUUV Guyuan strain.

A total of 725 specimens from all five organs (lung, liver, kidney, spleen, and gut) were subjected to RT-qPCR testing. PUUV was detected in all five organs of the same specimen, which was molecularly identified as *Myodes rufocanus* (family *Cricetidae*, genus *Myodes*), with a positive rate of 0.69% (1/145). The rodent species sequence is presented in S2 File. Among these organs, the lung showed the highest viral load (lowest CT value), followed by the kidney, spleen, and liver, while the gut exhibited the lowest viral load (highest CT value) (Fig 1A).

Fifteen pairs of specific primers were designed to amplify the complete genomic sequences of PUUV. Capillary electrophoresis analysis showed that full-genome amplification products of the PUUV exhibited a single peak at the expected size, confirming their specificity (Fig 1B). The amplification products were further validated through Sanger sequencing. The complete sequences of the L, M, and S segments—with respective lengths of 6547, 3680, and 1883 nucleotides—exhibited only 81.2%, 80.2%, and 84.3% nucleotide sequence identity with previously characterized reference viral strains Kitahiyama128L (AB712372), Tobetsu_04 (LC790462), and Baltic/205 Cg (AJ314599), respectively. Analyses of the deduced amino acid sequence homology revealed higher similarities reaching 95.4%, 94.6%, and 97.0% respectively. Based on a recently proposed quantitative framework for PUUV genotypic differentiation, viruses with less than 10% nucleotide divergence in the S segment are assigned to the same genotype [26]. A nucleotide divergence of 15.7%–19.8% across the three segments indicates that these sequences are likely derived from a novel genotype of PUUV. Based on its discovery location, the newly identified PUUV was named Guyuan strain.

### Viral isolation and detection

Given that the virus was first discovered in Hebei Province, we attempted to isolate it by inoculating the various positive samples (including lung, liver, kidney, and spleen tissues) into cell cultures. However, virus isolation attempts in Vero, Vero-E6, BHK-21 and A549 cell lines were unsuccessful. Viral nucleic acids were identified in the supernatant of Vero cell cultures at 7 days post-inoculation (dpi), but subsequent analysis of washed cells showed negative results. Continuous culture for 21 days with three blind passages (each spanning 21 days) yielded negative results. Similarly, lung, liver, kidney, and spleen samples inoculated into Vero, Vero-E6, BHK-21 and A549 cells tested negative at 7, 14, 21, 42, and 63 days post-inoculation. After blindly passaging the cells for three generations, the detection remained negative.

### Virus confirmation by transmission electron microscopy and indirect immunofluorescence assay

**Fig 2:**
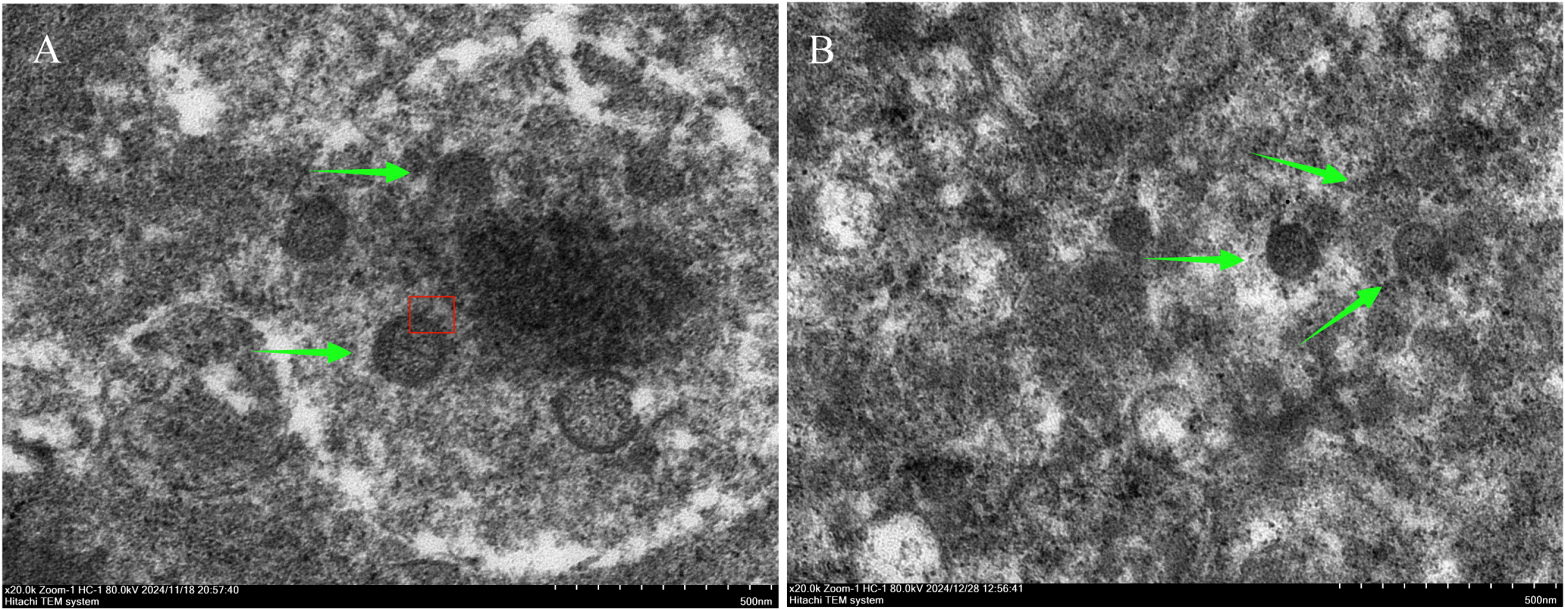
Morphology of the PUUV Guyuan strain by transmission electron microscopy. A: Green arrows indicate the virus particles in lung tissue, showing spherical morphology with a lipid envelope. The red box highlights a region of spike protein, and the electron-dense interior represents the ribonucleoprotein core; B: Green arrows indicate the virus particles in kidney tissue, with consistent spherical or oval morphology and a lipid envelope as observed in lung tissue. Scale bar = 50 nm. The scale bars are located in the lower-right corner of the images.

Owing to the failure to isolate Guyuan strain in cell culture, we sought to directly observe the virus in positive specimens. Transmission electron microscopy (TEM) of lung and kidney samples revealed detailed characterization of viral morphology. TEM imaging revealed intact virus particles of the Guyuan strain with a diameter of 90–140 nm, localized in the cytoplasm of pulmonary cells and renal cells. These particles exhibited a spherical morphology with a distinct lipid envelope and surface spike proteins, along with an electron-dense interior indicative of the ribonucleoprotein core. These features align with the typical structural characteristics of orthohantaviruses [27,28] (Fig 2).

**Fig 3:**
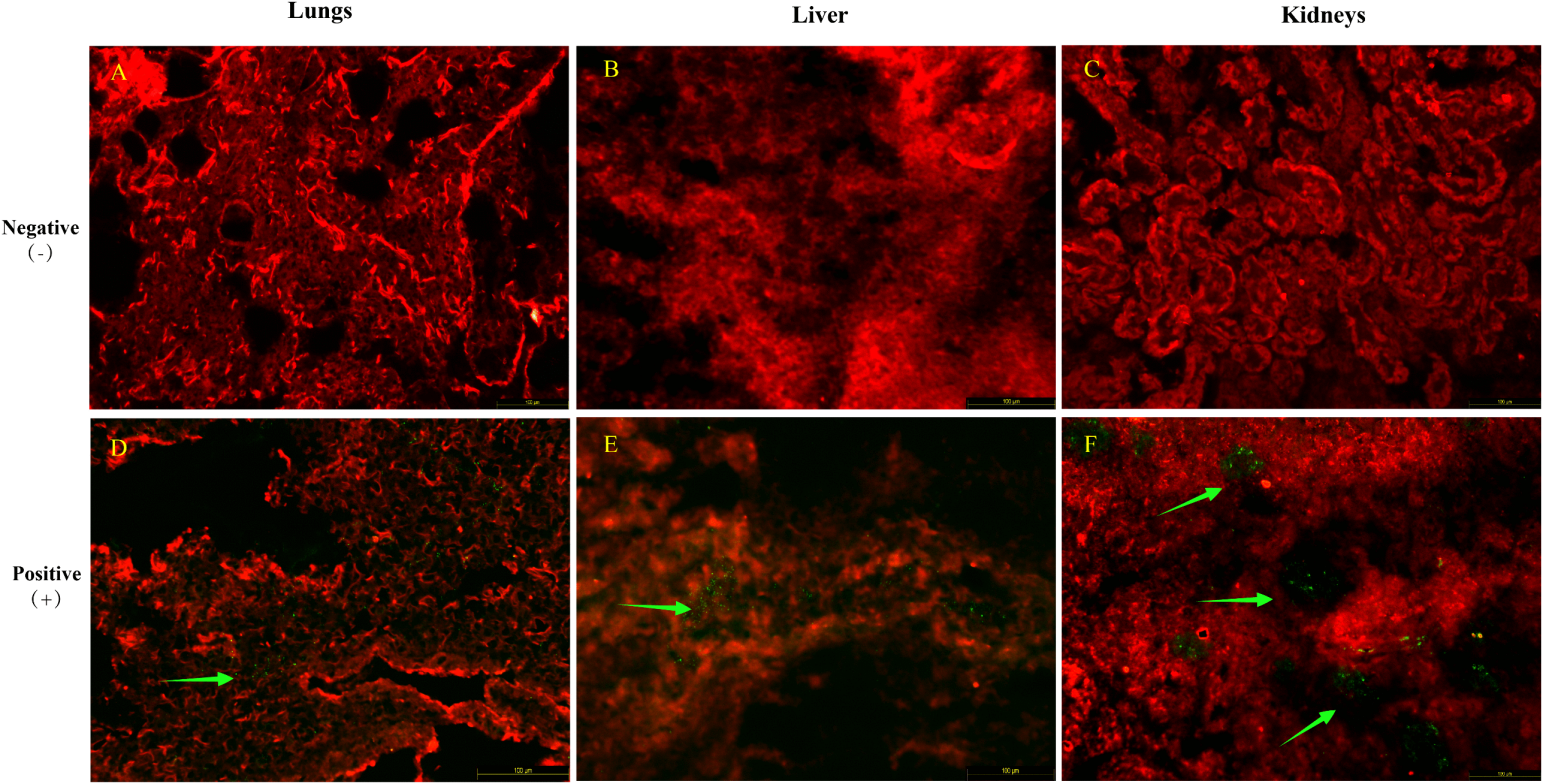
The PUUV Guyuan strain in the organs (lungs, liver, kidney) observed by immunofluorescence microscopy. A–C: Negative control tissues for the PUUV Guyuan strain (A: lung; B: liver; C: kidney). D–F: Positive tissues for the PUUV Guyuan strain (D: lung; E: liver; F: kidney). Green arrows indicate that granular fluorescence is indicative of PUUV protein antigenic presence. Scale bar = 100 μm and is located in the lower left.

Since Guyuan strain belongs to the family *Hantaviridae* and represents a distinct clade of PUUV, we characterized viral antigen distribution. An anti-Hantavirus Puumala mouse monoclonal antibody (clone A1C5, culture supernatant, Progen Biotechnik GmbH, Germany) was applied to detect PUUV antigen in sections of multiple organ tissues. Immunofluorescence staining demonstrated specific green fluorescence in the lung, liver and kidney, indicating the existence of viral protein antigen and confirming the multiorgan tropism of the orthohantavirus, which is consistent with its replication patterns. Compared with the lung and liver, the kidney exhibited more abundant and brighter green fluorescent particles, predominantly distributed in the glomeruli (Fig 3).

### Molecular characterization of Guyuan strain and its host

**Fig 4:**
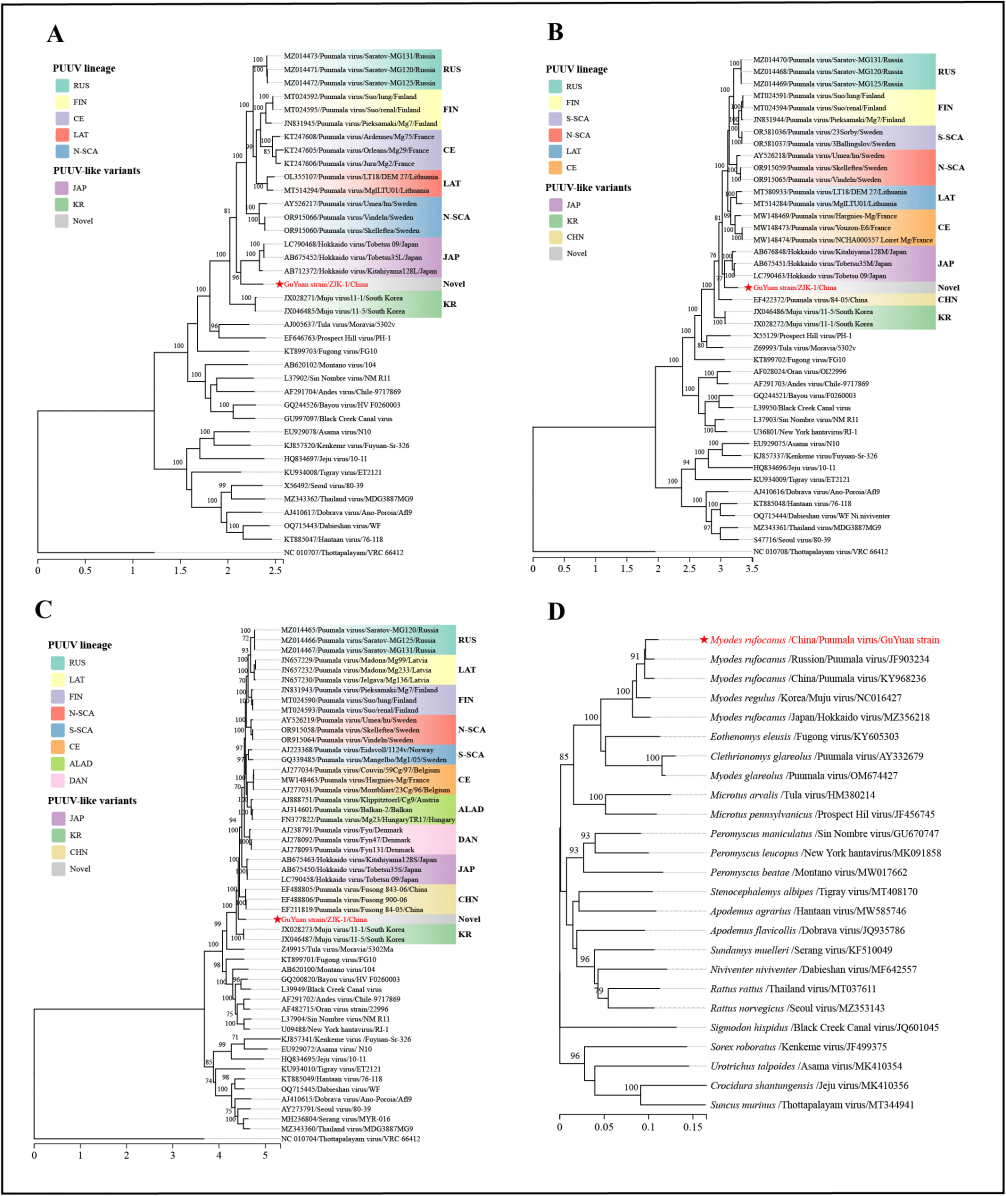
Phylogenetic analysis of Guyuan strain from *Myodes rufocanus.* **(A)** Phylogenetic tree constructed based on the full-length L segment using the ML approach, with the optimal substitution model GTR + F + I + G4; **(B)** Phylogenetic tree constructed based on the full-length M segment using the ML approach, with the optimal substitution model GTR + F + I + G4; **(C)** Phylogenetic tree constructed based on the full-length S segment using the ML approach, with the optimal substitution model TIM2 + F + I + G4; **(D)** Phylogenetic tree constructed based on the mitochondrial COI gene with the optimal substitution model TIM2 + F + I + G4. Red stars indicate the sequences of Guyuan strain from *Myodes rufocanus*; Scale bar indicates nucleotide substitutions per site.

The sequences of the L (Accession: PX068504), M (Accession: PX068503), and S (Accession: PX068502) segments of Guyuan strain were obtained via Sanger sequencing. Phylogenetic analysis incorporated 25 *Hantaviridae* members from the ICTV directory, encompassing 38 L, 42 M, and 53 S nucleic acid sequences. Phylogenetic trees were constructed using the ML approach with the corresponding optimal models: GTR + F + I + G4 (for L and M segments) and TIM2 + F + I + G4 (for the S segment). Phylogenetic analysis showed that the L, M, and S segments of Guyuan strain clustered within the same clade as those of PUUV and PUUV-like strains, while forming a distinct and independent subclade. Specifically, the L and M segments exhibited a closer genetic affinity to those of PUUV Hokkaido strain in Japan, whereas the S segment displayed a distinct, independent branch in the phylogenetic tree (Fig 4).

Among the 25 *Hantaviridae* members, homology analysis revealed that the L, M, and S nucleotide sequences of Guyuan strain exhibited the highest sequence identities with PUUV and PUUV-like strains, as follows: the L segment showed 79.2-80.5% identity, the M segment 78.0-80.5%, and the S segment 81.7-83.4%, which were significantly higher than those with other *Hantaviridae* members (Table 2). Amino acid sequence homology analysis further demonstrated that Guyuan strain exhibited the highest sequence identity with PUUV Hokkaido strain among all *Hantaviridae* members, particularly when compared to other lineages of PUUV. Specifically, the L, M, and S segments of Guyuan strain shared the highest amino acid identities of 95.4%, 94.6%, and 97.0% with PUUV Hokkaido strain, respectively (Table 2). Sequence alignment analysis revealed that the Guyuan strain displayed nucleotide divergences of 19.5%–20.8% in the L segment, 19.5%–22.0% in the M segment, and 16.6%–18.3% in the S segment when compared to other strains of the PUUV. Phylogenetic analysis further substantiated that the Guyuan strain constitutes a distinct and independent subclade. These genetic characteristics provide compelling evidence that the Guyuan strain represents a novel genotype of PUUV.

**Table 2:** Homology analysis of nucleotide and amino acid sequences of coding sequences (CDS) for the L, M, and S segments of Guyuan strain.

	L segment		M segment		S segment	
Guyuan strain	AA	nt	AA	nt	AA	nt
Puumala orthohantavirus/Fusong84–05/CHN	–	–	94.4	77.9	95.5	83.5
Puumala orthohantavirus/ALAD	–	–	–	–	94.5	83.0
Puumala orthohantavirus/CE	94.2	80.5	–	–	92.6	81.7
Puumala orthohantavirus/FIN	93.9	80.2	91.1	78.8	95.3	83.0
Puumala orthohantavirus/DAN	–	–	–	–	94.7	82.2
Puumala orthohantavirus/LT	94.1	80.1	91.7	78.8	95.3	83.3
Puumala orthohantavirus/N-SCA	93.5	79.7	91.2	79.8	95.4	83.0
Puumala orthohantavirus/S-SCA	–	–	–	–	94.2	83.2
Puumala orthohantavirus/RUS	93.8	79.9	92.1	79.1	94.7	83.2
Hokkaido orthohantavirus/JAP	95.4	80.3	94.6	80.5	97.0	82.9
Muju orthohantavirus/KR	92.6	79.2	92.9	78.0	94.9	81.7
Andes orthohantavirus	77.0	70.7	66.0	65.2	70.0	74.8
Asama orthohantavirus	65.9	64.2	51.6	57.3	58.6	61.5
Bayou orthohantavirus	77.8	70.9	64.2	63.6	74.1	70.2
Black Creek Canal orthohantavirus	77.7	71.0	64.7	64.5	74.3	70.6
Dabieshan orthohantavirus	69.3	65.8	68.5	75.2	60.6	64.4
Dobrava_orthohantavirus	69.9	66.7	54.3	58.6	61.3	62.1
Fugong orthohantavirus	79.0	72.5	71.3	69.2	75.2	71.4
Hantaan orthohantavirus	69.3	66.1	53.6	58.4	61.1	62.2
Jeju orthohantavirus	67.8	65.5	52.4	57.9	59.1	62.1
Kenkeme orthohantavirus	68.7	66.2	52.3	57.6	59.1	61.9
Montano orthohantavirus	76.9	70.9	66.1	65.0	70.3	72.9
New York orthohantavirus	–	–	66.7	65.2	72.4	68.5
Oran orthohantavirus	–	–	65.8	64.8	73.8	69.8
Prospect Hill orthohantavirus	83.1	72.8	76.4	70.6	80.8	74.8
Seoul orthohantavirus	68.9	66.1	53.3	58.3	63.9	62.7
Serang orthohantavirus	–	–	–	–	62.2	64.1
Sin Nombre orthohantavirus	77.4	70.9	67.3	66.2	71.0	69.0
Tula orthohantavirus	86.1	75.1	79.2	72.0	80.9	74.4
Thailand orthohantavirus	69.1	66.2	53.3	57.9	61.8	63.3
Tigray orthohantavirus	68.7	66.3	53.0	57.6	61.2	63.0
Thottapalayam orthohantavirus	62.7	62.4	42.1	50.6	46.4	55.0

To infer the evolutionary relationships between Guyuan strain and its host, a ML tree was generated under the optimal substitution model TIM2 + F + I + G4, using the mitochondrial COI gene from Rodentia hosts of representative orthohantaviruses, with *Soricomorpha* hosts serving as the outgroup (Fig 4D). The host phylogenetic tree was congruent with findings from prior studies, where the *Murinae* subfamily and *Cricetidae* family clustered into two monophyletic clades, with *Soricomorpha* as the outgroup. The *Myodes rufocanus* from which Guyuan strain was isolated exhibits a close phylogenetic relationship with Cricetinae host species such as *Myodes glareolus*, *Myodes regulus,* and *Clethrionomys glareolus,* which harbor other Puumala orthohantaviruses.

## Discussion

Over the past two decades, orthohantaviruses have emerged as globally concerning zoonotic pathogens [29]. Although identified in rodents, insectivores, and bats, only rodent-borne orthohantaviruses are confirmed human pathogens, causing two distinct diseases: HFRS in Asia and Europe, and HPS in the Americas. HFRS has been endemic in China for several decades and remains a significant zoonotic disease caused by orthohantaviruses. HFRS emerged in China in the early 1930s, and as of now, 29 out of 31 provinces have reported epidemics of this zoonotic disease [30,31]. Although previous studies have identified the presence of HTNV, SEOV, Dabieshan virus [32], PUUV-like virus [12], Amur virus [33], Luxi virus [34], Fugong virus [3], and TULV [35] in China, the primary etiological agents responsible for HFRS in the country are predominantly HTNV and SEOV. Liu [36] and Tang [37] respectively documented the presence of PUUV-related orthohantavirus in *Clethrionomys rufocanus* from Northeast China and in *Myodes rutilus* from Inner Mongolia in the early 21st century. To date, no additional detections have been reported in other geographic regions in China. This study detected a distinct genotype of PUUV in *Myodes rufocanus* from Bashang Grassland, Hebei Province, through metagenomic and Sanger sequencing analyses.

Given that the isolation of the novel PUUV strain is necessary to enhance our understanding of this pathogen, attempts were made to isolate it from various virus-positive organ tissues using cell lines including Vero, Vero E6, BHK-21 and A549 cells. Despite three rounds of blind passage, all isolation attempts were unsuccessful. These results underscore the inherent challenge in cultivating PUUV and other Arvicolinae-borne hantaviruses in cell cultures [38]. Orthohantaviruses present significant challenges for isolation via conventional cell culture systems, primarily due to their slow replication rates and non-cytopathic nature, which culminates in a low recovery rate of the viruses [39–41]. Previous research has demonstrated that rodent samples with persistent orthohantavirus infections may contain neutralizing antibodies that can neutralize viral particles during the isolation process [38]. Therefore, utilizing animals that test positive for hantavirus antigens and RNA but are seronegative could potentially enhance the success rate of viral isolation. Furthermore, given the relatively strict one-host-one-virus specificity characteristic of hantaviruses, the efficiency of viral isolation can be improved by employing cell lines derived from the natural rodent hosts of the specific hantaviruses being targeted [41].

Previous studies have demonstrated that SEOV exhibits broader multiorgan tropism [42], which is attributed to hantavirus infection-induced multiorgan involvement via mechanisms including direct vascular endothelial cell damage, dysregulated immune responses, and microcirculatory disturbances [43]. In our study, the novel PUUV was detected in five organs (lung, liver, kidney, spleen, and gut) of the same specimen, with the lung displaying the highest viral load. This observation aligns with previous studies that have predominantly focused on HV detection in lung tissues, recognized as primary sites of viral replication [44]. Fig 2 provides direct visual evidence for the presence of viral particles within both pulmonary and renal tissues of the infected hosts. Moreover, immunofluorescence staining of different organs in Fig 3 revealed that the kidney exhibited more abundant and brighter green fluorescent particles, which were predominantly distributed in the glomeruli compared to other organs, indicating that the kidney is a crucial target organ for viral replication [45].

Phylogenetic analysis of Guyuan strain sequences revealed that the L and M segments displayed a closer genetic affinity to their counterparts in PUUV Hokkaido strain from Japan, whereas the S segment exhibited a distinct and independent branch in the phylogenetic tree. The topological discrepancy may be attributed to differences in evolutionary rates and varying selection pressures acting on the genomic segments [46], but also to possible reassortment events [47], a recognized evolutionary mechanism in tri-segmented orthohantaviruses that can result in incongruent phylogenetic relationships among the L, M, and S segments. This analysis further indicated genetic diversity as well as geographic clustering of genetic variants of PUUV [48]. Moreover, the L, M, and S segments of Guyuan strain shared the highest amino acid identities with PUUV Hokkaido strain, indicating that the evolutionary trajectory of PUUV in China is more closely aligned with that in Japan than with those in regions such as Europe and Russia. Phylogenetic and amino acid homology analyses suggest that the Guyuan strain and PUUV Hokkaido strain from Japan likely diverged from a common ancestor virus.

Typically, each orthohantavirus species tends to exhibit a primary association with a single host species [49]. For instance, HTNV has been identified in *Apodemus agrarius* across the Russian Far East, China, and South Korea. Meanwhile, SEOV, which is harbored by *Rattus norvegicus*, exhibits a global distribution [50]. In the present study, the host animal *Myodes rufocanus* harboring the PUUV Guyuan strain, together with other cricetid host species including *Myodes glareolus, Myodes regulus*, and *Clethrionomys rufocanus*, all belong to the genus *Myodes* within the subfamily Arvicolinae. This taxonomic congruence between the *Myodes* host species and their associated PUUV further supports long-term co-evolution of host and virus, whereby each typically maintains stable transmission exclusively within its primary mammalian host species or closely related species [7]. Subsequent studies should analyze PUUV multi-segment genomic data to clarify its evolutionary patterns and driving forces.

There are some limitations in this study. Firstly, the PUUV Guyuan strain was identified in only one sample. *Myodes rufocanus*, the natural reservoir of PUUV, is not a dominant wild rodent species in the study area, resulting in extremely limited captures of this species during the investigation. The scarcity of comprehensive PUUV-positive rodents is inadequate to accurately reflect the local prevalence and distribution of the pathogen, which hinders a holistic assessment of the trends in PUUV prevalence in Hebei Province. Secondly, this study has not yet conducted a dedicated survey on PUUV infection among the local healthy population, resulting in a lack of data on the PUUV infection rate and transmission patterns in the local population. Consequently, a scientific and accurate assessment of the spillover transmission risk of PUUV from infected animal hosts to humans cannot be performed.

In summary, a distinct genotype of PUUV was identified in *Myodes rufocanus* from Bashang Grassland in Guyuan County, China. Phylogenetic analysis indicated that the sequences exhibited the highest identity to PUUV Hokkaido strain in Japan. Viral particles were observed in pulmonary and renal cells by TEM, and viral protein antigen was detected in viral RNA-positive lung, liver, and kidney tissues by IFA, confirming the virus’s multiorgan tropism. Further studies are necessary to establish longitudinal surveillance of rodent populations to clarify the transmission dynamics and assess the pathogenicity of the PUUV. Additionally, targeted assessments of viral load in local human populations should be conducted to evaluate the risk of zoonotic spillover. These efforts will provide more robust evidence for comprehending the virus’s impacts on human health and guiding subsequent prevention and control strategies.

## Supporting information

S1 Table Primers for full-genome amplification of Guyuan strain. (DOCX)

S1 File Animal tissue TEM staining protocol. (DOCX)

S2 File Mitochondrial COI gene sequence of *Myodes rufocanus.* (DOCX)
