# Supplementary material for: Identification and genetic characterization of a distinct genotype of Puumala orthohantavirus in Hebei Province, China
Source: PLoS Negl Trop Dis. 2026 May 11;20(5):e0014250. doi: 10.1371/journal.pntd.0014250 (PMC13175471; doi:10.1371/journal.pntd.0014250)
Supplement: S1 Table — (DOCX) [file pntd.0014250.s001.docx]

**S1 Table.** **Primers for full-genome amplification of the Guyuan strain of Puumala virus**

| **Primer** | **Locate** | **Sequence（5’→3’）** | **Target** |
| --- | --- | --- | --- |
| S1F | 1-20 | TAGTAGTAGACTCCTTGAAG |  |
| S1R | 387-404 | CTATACTGTCTGGTGAAG |  |
| S2F | 78-95 | ACGCCATGAGCAACAACT | S segment |
| S2R | 1141-1158 | ACGCCTTAGGTAGGACTG |  |
| S3F | 858-876 | CAGGCAAGATGTGTTAGAC |  |
| S3R | 1861-1883 | TAGTAGTATGCTCCTTGAAAAGC |  |
| M1F | 1-25 | TAGTAGTAGACTCCGCAAGAAGAAG |  |
| M1R | 588-605 | CTAACTGACCAGATACAC |  |
| M2F | 192-209 | TGCCACCTGTTCCTCTTA |  |
| M2R | 958-975 | GCTTTGCCTGTGACCTTA |  |
| M3F | 958-975 | TAAGGTCACAGGCAAAGC | M segment |
| M3R | 2374-2391 | CCAACTCCAGGGCAATCT |  |
| M4F | 2152-2169 | TGCTAATGGGCAAGAACA |  |
| M4R | 3492-3509 | TCAACAAGCCTGATGGAA |  |
| M5F | 2873-2890 | GCAAGTGCGCTTGAATGG |  |
| M5R | 3651-3670 | CTCCGCAGGAACAAAAGTCC |  |
| L1F | 1-23 | TAGTAGTAGACTCCGAGATAGAG |  |
| L1R | 971-989 | CAAGCCAAACGTGTATCAT |  |
| L2F | 865-882 | ATACATGCCTTACGCTAC |  |
| L2R | 1808-1825 | GAGGGAAATGACCTTGAT |  |
| L3F | 1587-1604 | ATTAGAGGTAGCAGGGTC |  |
| L3R | 2613-2630 | ACTGTCGGGTTAGTTTGA |  |
| L4F | 2502-2521 | TAGCCAGACAAGAAACATTA | L segment |
| L4R | 3764-3783 | ATTATTCACCATACCAGGAG |  |
| L5F | 3697-3714 | TGCCCACAGTTAGCACAG |  |
| L5R | 4942-4959 | AGCCTTACACCATCTCCA |  |
| L6F | 4711-4728 | GTTGCTCCGCTCCTGTAT |  |
| L6R | 5959-5978 | GTTTCATCATCATCGCTGTC |  |
| L7F | 5560-5577 | AGAGCAATGGCACAACTC |  |
| L7R | 6526-6547 | TAGTAGTATGCTCCGAGAAAAG |  |
