# Supplementary material for: Identification and genetic characterization of a distinct genotype of Puumala orthohantavirus in Hebei Province, China
Source: PLoS Negl Trop Dis. 2026 May 11;20(5):e0014250. doi: 10.1371/journal.pntd.0014250 (PMC13175471; doi:10.1371/journal.pntd.0014250)
Supplement: S1 File — (DOCX) [file pntd.0014250.s002.docx]

**Animal tissue TEM staining protocol**

# Apparatus and reagents

- 1. **Major apparatus**

**Name Producer Model**

Ultra microtome Leica Leica UC7

Diamond slicer Daitome Ultra 45°

Transmission Electron Microscope HITACHI HT7800/HT7700

150 meshes cuprum grids with formvar film

Servicebio WFHM-150

# Major reagents

**Name Producer Code**

Fixative for TEM Servicebio G1102

Ethanol Sinaopharm Group Chemical Reagent Co. LTD 100092183

Aceton Sinaopharm Group Chemical Reagent Co. LTD 10000418

EMBed 812 SPI 90529-77-4

OsO_4_ Ted Pella Inc 18456

# Procedure

- 1. **Harvest tissue block and fixation**: Targeted fresh tissues should be selected to minimize mechanical damage such as pulling, contusion and extrusion. Use a sharp blade to cut and harvest fresh tissue blocks quickly within 1-3 minutes. The size of tissue block should be no more than 1 mm3. Before sampling, petri dishes with fixative for TEM should be prepared in advance, small tissue blocks could be removed from animal body and immediately put into petri dishes, and then cut into small size of 1 mm3 in the fixative. The 1mm3 tissue blocks were transferred into an EP tube with fresh TEM fixative for further fixation, which was fixed at 4℃ for preservation and transportation. And then wash the tissues using 0.1 M PB (pH 7.4) for 3 times, 15 min each.
  2. **Post-fix:** Tissues avoid light post fixed with 1% OsO_4_ in 0.1 M PB (pH 7.4) for 2 h at room temperature. After remove OsO_4_, the tissues are rinsed in 0.1 M PB (pH 7.4) for 3 times, 15 min each.

# Dehydrate at room temperature as followed:

30% ethanol for 20 min;

50% ethanol for 20 min;

70% ethanol for 20 min;

80% ethanol for 20 min;

95% ethanol for 20 min;

Two changes of 100% ethanol for 20 min; Finally two changes of acetone for 15 min.

# Resin penetration and embedding as followed:

Acetone：EMBed 812=1:1for 2-4 h at 37℃; Acetone：EMBed 812=1:2 overnight at 37℃; pure EMBed 812 for 5-8 h at 37℃;

Pour the pure EMBed 812 into the embedding models and insert the tissues into the pure EMBed 812, and then keep in 37℃ oven overnight.

- 1. **Polymerization:** The embedding models with resin and samples were moved into 65℃ oven to polymerize for more than 48h. And then the resin blocks were taken out from the embedding models for standby application at room temperature.
  2. **Ultrathin section:** The resin blocks were cut to 60-80nm thin on the ultra microtome, and the tissues were fished out onto the 150 meshes cuprum grids with formvar film.
  3. **Staining:** 2% uranium acetate saturated alcohol solution avoid light staining for 8 min, rinsed in 70% ethanol for 3 times and then rinsed in ultra pure water for 3 times. 2.6% Lead citrate avoid CO_2_ staining for 8 min, and then rinsed with ultra pure water for 3 times. After dried by the filer paper, the cuprum grids were put into the grids board and dried overnight at room temperature.
  4. **Observation and images capture:** The cuprum grids are observed under TEM and take images.
