# Supplementary material for: Identification and genetic characterization of a distinct genotype of Puumala orthohantavirus in Hebei Province, China
Source: PLoS Negl Trop Dis. 2026 May 11;20(5):e0014250. doi: 10.1371/journal.pntd.0014250 (PMC13175471; doi:10.1371/journal.pntd.0014250)
Supplement: S2 File — (DOCX) [file pntd.0014250.s003.docx]

**Mitochondrial COI gene sequence of *Myodes rufocanus***

TCGCTGATTATTCTCTACCAATCACAAGGACATCGGGACCTTGTATCTCCTATTTGGGGCCTGAGCAGGAATAGTAGGGACAGCCCTTAGCATCCTAATCCGAGCGGAACTTGGGCAACCAGGCGCCCTATTAGGTGATGACCAGATCTACAATGTAGTAGTTACAGCTCACGCATTTGTCATAATTTTCTTCATAGTGATGCCAATGATAATTGGCGGGTTCGGTAACTGACTAGTCCCATTAATAATTGGGGCCCCTGATATGGCATTCCCACGAATAAATAATATGAGCTTCTGACTCCTACCCCCATCATTCCTTCTCCTCCTAGCATCATCCATAGTAGAAGCAGGAACTGGAACAGGATGAACTGTTTACCCCCCATTAGCCGGTAATTTAGCACATGCAGGAGCATCCGTAGACTTAACCATCTTTTCTCTTCACCTAGCAGGGGTCTCCTCAATTCTAGGGGCTATTAATTTTATCACTACAATTATCAACATAAAACCACCAGCTATAACGCAATACCAAACACCTCTATTTGTATGATCAGTATTAATTACAGCCGTACTCCTCCTCCTTTCTCTTCCAGTATTAGCCGCAGGTATTACAATGCTCCTCACCGATCGAAACCTAAACACCACTTTCTTTGACCCGGCCGGAGGTGGCGACCCTATTTTATACCAACACTTATTCTGATTCTTTGGACAC
